# Implant Removal Complications in Pediatric Orthopedics

**DOI:** 10.1055/s-0045-1814112

**Published:** 2025-12-30

**Authors:** Marcela de Andrade Balsano, Heloisa Zimmermann Faggion, Alexander Cordeiro Bornhold, Weverley Rubele Valenza, Jamil Faisal Soni

**Affiliations:** 1 Hospital do Trabalhador, Curitiba, PR, Brazil; 2 Hospital Universitário Cajuru, Curitiba, PR, Brazil

**Keywords:** device removal, orthopedics, pediatrics, postoperative complications, complicações pós-operatórias, ortopedia, pediatria, remoção de dispositivo

## Abstract

**Objective:**

Implant removal is a common practice in pediatric orthopedics, despite its risks. The present study aims to evaluate postoperative complications following implant removal in pediatric patients, correlating them with epidemiological factors.

**Methods:**

Retrospective cross-sectional study, conducted in a tertiary hospital, with analysis of medical records and imaging exams from February 2021 to June 2024. Medical records of patients under 18-years-old who were followed up until outpatient discharge were evaluated. The research included age, sex, type of implant, indication for insertion and removal, time to implant removal, and postoperative complications, which were classified according to Clavien-Dindo.

**Results:**

A total of 202 medical records were analyzed. Implant removal was more common in boys, with a mean age of 12 years, and the mean time to removal was 16 months. The main reason for placement was orthopedic trauma, and for removal, bone consolidation. The complication rate was 10% (n = 22). Plate removal had the highest complication rate (15%), followed by isolated screws (14%), external fixators (12%), flexible nails (10%), and Kirschner wires (8%). The main complications were unsuccessful removal (45.5%), superficial infection (36.5%), refractures (9%), and movement limitation (9%). The Clavien-Dindo classification revealed 45.45% type I complications, 40.9% type II complications, and 13.6% type IIIa complications.

**Conclusion:**

Implant removal in pediatric orthopedics is not without complications, with 11% being found in this study. Failure to completely remove the implant, superficial infections, and refractures were the most common. Before the procedure, the risks and benefits involved should be considered and consensus should be reached among family members and surgeons.

## Resumo

**Objetivo:**

A remoção de implantes é uma prática comum na ortopedia pediátrica, apesar de seus riscos. Este estudo visa avaliar as complicações pós-operatórias da remoção de implantes em pacientes pediátricos, correlacionando-os com fatores epidemiológicos.

**Métodos:**

Estudo transversal retrospectivo, realizado em um hospital terciário, com análise de prontuários e exames de imagem entre fevereiro de 2021 e junho de 2024. Foram avaliados prontuários de pacientes menores de 18 anos, acompanhados até a alta ambulatorial. A pesquisa incluiu idade, sexo, tipo de implante, indicação da inserção e retirada, tempo de permanência do implante e complicações pós-operatórias, que foram classificadas de acordo com Clavien-Dindo.

**Resultados:**

Foram analisados 202 prontuários. A retirada de implantes foi mais comum em meninos, com média de idade de 12 anos e tempo médio de permanência de 16 meses. O principal motivo da colocação foi trauma ortopédico, e o da remoção, consolidação óssea. A taxa de complicações foi de 10% (n = 22). A remoção de placas teve a maior taxa de complicações (15%), seguida por parafusos isolados (14%), fixadores externos (12%), hastes flexíveis (10%) e fios de Kirschner (8%). As principais complicações foram retirada malsucedida (45,5%), infecção superficial (36,5%), refraturas (9%) e limitação de movimento (9%). A classificação de Clavien-Dindo revelou 45,45% de complicações tipo I, 40,9% tipo II e 13,6% tipo IIIa.

**Conclusão:**

A remoção de implantes dentro da ortopedia pediátrica não é isenta de complicações, sendo encontradas 11% neste estudo. A retirada completa malsucedida do implante, infecções superficiais e refraturas foram as mais comuns. Antes do procedimento, os riscos e benefícios envolvidos devem ser considerados, sendo necessário um consenso com familiares e cirurgiões.

## Introduction

Implant removal after fracture consolidation is a prevalent procedure in orthopedic practice. 1 It is estimated that 6% of all orthopedic procedures are implant removals, and in the pediatric population, this rate can reach 6.7%. 2

There is still no clinical guideline to determine the indication and timing of implant removal, making this a topic of discussion in the orthopedic community. 3

The benefits of removing the implants include the prevention of biological and functional sequelae, such as tumor induction, infection, inflammation, and, if necessary, an easier way to perform reconstructive surgeries. 4

Implant removal is not a procedure without risks and complications, so its indication should be discussed with those responsible preoperatively. During removal, there is a risk of damage to neurovascular structures, broken screws, impossibility of complete material removal, need to enlarge the initial incision or make new ones, evolution with superficial or deep infection, and increased risk of refracture. 3 The risk of complications associated with implant removal reported in the literature is approximately 10%. 5

There is no evidence in the current literature to fully support or refute routine implant removal in children. Therefore, the study of implant removal is interesting due to its impact on orthopedic practices and health care costs. 3

This study aims to analyze postoperative complications in implant removal of pediatric patients, correlating them with epidemiological factors and type of removed implant.

## Materials and Methods

This is a retrospective cross-sectional study, based on medical records and imaging exams from a tertiary hospital, from February 2021 to June 2024.

This study was submitted to the Research Ethics Committee and approved under the number CAAE: 83355224.6.0000.5225.

Patients under 18-years-old who underwent surgery for implant removal were analyzed. They were followed up until outpatient discharge and had their medical records fully completed, with a sample estimate of 350 patients.

Those who had exposed hardware, who underwent outpatient implant removal, those with incomplete medical records, and those who lost postoperative follow-up, were excluded.

The variables collected for analysis were age, sex, type of implant used, surgical indication for insertion (trauma vs. orthopedic disease), implant insertion site (upper vs. lower limb), indication for removal, time to implant removal, and complications related to removal.

Postoperative complications related to implant removal were considered in this study: failure to remove the implant, refractures, limitation of range of motion in the postoperative period, and superficial surgical wound infections.

For the analysis of the postoperative complications, the Clavien-Dindo classification (CDC) was used. 6 7 In type I, any deviation from the ideal postoperative course without the need for pharmacological treatment or surgical and/or radiological interventions. Type II patients require pharmacological treatment with drugs other than those allowed for type I complications. Type III requires surgical and/or radiological intervention, divided into (a) without and (b) with general anesthesia. Type IV refers to life-threatening complications, including (a) single and (b) multiple organ dysfunction. Finally, type V refers to patient death. 7

Data were quantitatively analyzed using Microsoft Excel 2010 (Microsoft Corp.) for absolute and relative frequency measurements. Comparisons between variables were performed using the Chi-squared test for quantitative variables. The statistical analyses were conducted in the R program (RStudio, 2020), and *p* -values < 0.05 were considered significant.

## Results

**Fig. 1:**
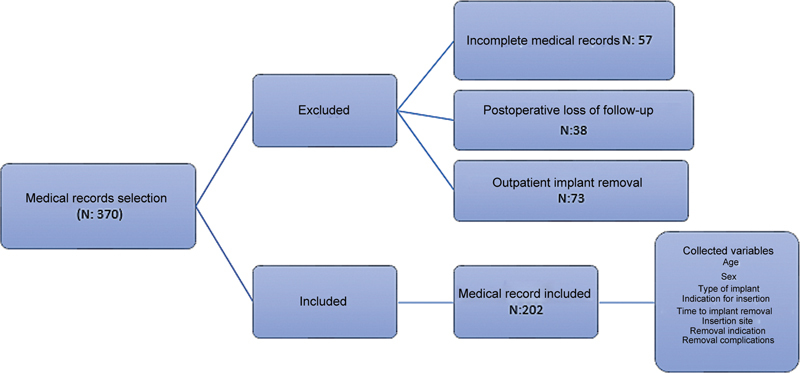
Flowchart of the selection of patients' medical records.

There were 370 medical records selected, with patients aged 0 to 18 years who underwent implant removal between February 2021 and June 2024. After applying the exclusion criteria, 202 were included in the sample analyzed ( Fig. 1 ).

Of the 202 patients in the sample, 156 (77.2%) were male and 46 (22.8%) were female. The mean age of the sample was 11.5 years, ranging from 4 to 17 years. In males, the mean age was 11.7 years; in females, 10.8 years.

The mean time to implant removal was 9.7 months, ranging from 1 to 72 months. Table 1 shows the mean time to implant removal in patients who presented complications (14.4 months) versus those who did not (10.3 months).

**Table 1:** Evolution time (months) according to complication in 202 patients

Complications	Mean evolution (months)	SD	*p* -value
No (n = 180)	10.3	9.0	0.846
Yes (n = 22)	14.4	16.7	

**Abbreviation:** SD, standard deviation.

Of the implants removed, 62 were Kirschner wires (intramedullary for forearm fracture fixation or transosseous fixation left buried subcutaneously), 70 flexible intramedullary nails, 17 external fixators, 41 plates and screws, and 8 isolated screws. The mean implant retention time until removal, in months, is shown in Table 2 .

**Table 2:** Time to implant removal according to the type of material inserted in 202 patients

Type of material inserted*	Mean time to implant removal (months)	SD
KW (n = 62)	6.5	5.6
External fixator (n = 17)	4.6	3.1
Intramedullary nails (n = 70)	11.0	6.8
Screw (n = 8)	14.3	11.5
Plate (n = 41)	18.5	15.8

**Abbreviations:** KW, Kirschner wires; SD, standard deviation.

**Note:** *Patients who had more than one type were removed.

The reason for implant removal in 83.6% (n = 169) of the patients was due to bone consolidation, discomfort and/or prominence related to the implant in 8.98% (18), having achieved the desired limb alignment (post-, hemi-, or epiphysiodesis) in 3.46% (n = 7), limitation of range of motion related to the implant in 1.98% (n = 4), exposure of the material in 1.48% (n = 3), and due to superficial infection related to the implant in 0.5% (n = 1).

Complications were reported in 11% (n = 22), among which 36.5% were superficial infection (8/22), 9% of temporary range of motion limitation after removal that recovered in outpatient follow-up (2/22), 9% of refractures (2/22), and 45.5% of failure in total implant removal (10/22), which was the most frequent one. There was a 10% rate in male and 13% in female patients.

Analyzing the complications related to the type of implant removed, 8% were in Kirschner wires (5/62 withdrawals), 12% in external fixators (1/17), 10% in flexible intramedullary nails (7/70), 14% in isolated screws (2/8), and 15% in plates and screws (7/41).

Regarding the indication for implant insertion, 24 were due to orthopedic disease, among whom 17% (n = 4), and 178 were patients with a traumatic incident, among whom 10% (n = 18) experienced complications.

Regarding the sites of implant removal, 100 were in the lower limb and 101 in the upper limb, with complication rates of 12 and 10%, respectively. The data analyzed of the total sample are shown in Table 3 .

**Table 3:** Presence or absence of complications according to different qualitative variables in 202 patients

Variable	Categories	Complications (%)		*p* -value
		No (n = 180)	Yes (n = 22)	
Sex	Female (n = 46)	87	13	0.595
	Male (n = 156)	90	10	
Implant*	KW (n = 62)	92	8	0.867
	External fixator (n = 17)	88	12	
	Flexible nails (n = 70)	90	10	
	Screw (n = 7)	86	14	
	Plate (n = 41)	85	15	
Reason for insertion	Disease (n = 24)	83	17	0.307
	Trauma (n = 178)	90	10	
Site of trauma	Lower limb (n = 100)	88	12	0.659
	Upper limb (n = 101)	90	10	

**Abbreviation:** KW, Kirschner wires.

**Note:** *Patients who had more than one category were removed.

Considering the sample only of patients who had complications ( Table 4 ), the mean age was 12 (6–17) years, the mean time to removal was 16 (2–72) months. Furthermore, 50% (11–22) occurred in the upper limbs, and 50% (11–22) in the lower ones.

**Table 4:** Postoperative complications after implant removal grouped according to epidemiological characteristics evaluated

Sex	Age (years)	Inserted material	Reason for insertion	Surgical site	Time to removal (months)	Reason for removal	Complications
F	6	Intramedullary nails	Femoral diaphyseal fractures	Thigh	7	Consolidation	Infection SW
M	9	Intramedullary nails	Femoral diaphyseal fractures	Thigh	8	Infection SW	Restricted ROM
M	9	Plate 2.7mm	Right hallux arthrodesis	Feet	10	Consolidation	Infection SW
M	9	Cancellous screw 3.5mm + washer	Pseudarthrosis of the lateral condyle	Elbow	7	ROM limitation	Unsuccessful removal
F	10	Angled hip plate	Paralytic hip dislocation	Hip	72	Exposed hardware	Infection SW
F	10	Dynamic compression plate 4.5mm	Distal tibial fracture	Leg	12	Consolidation	Unsuccessful removal
F	11	External fixator	Distal femoral fracture	Thigh	7	Consolidation	Refracture
M	11	Dynamic compression plate 4.5mm	Subtrochanteric fracture	Thigh	12	Consolidation	Unsuccessful removal
M	11	Intramedullary nails	Femoral diaphyseal fractures	Thigh	7	Consolidation	Infection SW
M	11	KW	Forearm fracture	Forearm	6	Consolidation	Infection SW
M	12	KW	Forearm fracture	Forearm	2	Exposed hardware	Infection SW
M	13	KW	Forearm fracture	Forearm	5	Consolidation	Restricted ROM
M	13	DCP 4.5mm	Diaphyseal fractures of the radius	Wrist	14	Consolidation	Unsuccessful removal
M	13	Intramedullary nails	Femoral diaphyseal fractures	Thigh	24	Consolidation	Unsuccessful removal
M	13	KW	Forearm fracture	Forearm	6	Consolidation	Infection SW
F	14	Cannulated screw 3.0	Medial epicondyle fracture	Elbow	31	Consolidation	Unsuccessful removal
M	14	DCP 4.5mm	Femoral diaphyseal fractures	Thigh	53	Consolidation	Unsuccessful removal
F	14	Intramedullary nails	Humerus diaphyseal fractures	Shoulder	10	Consolidation	Unsuccessful removal
M	14	KW	Forearm fracture	Forearm	5	Consolidation	Refracture
M	15	Intramedullary nails	Proximal humerus fractures	Shoulder	16	Material discomfort	Unsuccessful removal
M	15	Plate in 8	Limb-length discrepancy	Knee	12	Alignment	Infection SW
M	17	Intramedullary nails	Forearm fracture	Forearm	24	Consolidation	Unsuccessful removal

**Abbreviations:** DCP, dynamic compression plate; KW, Kirschner wires; ROM, range of motion; SW, surgical wound; TEM,.

Analyzing the anatomical site in which the complications occurred: 7 implants were removed from the thigh, 6 from the forearm, 2 from the elbow, 2 from the shoulder, 1 from the hip, 1 from the knee, 1 from the leg, 1 from the foot, and 1 from the elbow.

Regarding the type of implant among cases with complications, there were 31.8% (n = 7) with flexible nails, 31.8% (n = 7) plates, 22.9% (n = 5) Kirchner wires, 9.0% (n = 2) isolated screws, and 4.5% (n = 1) with external fixators.

Analyzing the reason for implant removal, 73% (n = 16) were due to bone consolidation, 9% (n = 2) implant exposure, 4.5% (n = 1) limited range of motion, 4.5% (n = 1) implant discomfort, and 4.5% (n = 1) local superficial infection.

Using the CDC, 6 7 there were 10 type I complications (45.45%), 9 type II (40.9%), and 3 type IIIa (13.6%), as shown in Table 5 .

**Table 5:** Postoperative complications after implant removal grouped accordingly to Clavien-Dindo classification

Type	N (%)
I	10 (45.45%)
II	9 (40.9%)
IIIa	3 (13.6%)
IIIb	0 (0)
IV	0 (0)
V	0 (0)

## Discussion

Implant removal is a relatively common procedure in pediatric orthopedics, especially when there are signs of infection, the implant causes discomfort, or it may alter bone growth. 4

Depending on the implant site, permanence may make future procedures difficult. 8 The advantages and disadvantages of removing implants in children are discussed, so studying the possible complications of these procedures may assist in decision-making. 4

The study by AlOmran et al *.* , 1 evaluated the routine implant removal in 167 patients, with a complication rate of 6%. Similarly, the study by Desai et al. 8 found a 9.5% complication rate after analyzing 2,176 cases. In our series, after evaluating 202 cases, we found an overall complication rate of 11%, which is consistent with the literature.

Also, according to Desai et al., 8 implant removal after a long time since insertion is associated with a higher risk of complications, especially incomplete removal or material breakage. In these situations, the implant may be covered by a bony callus that forms over time, prolonging the time required for its removal and potentially leading to incomplete removal, making the procedure more invasive and increasing the risk of complications. 4 9

The higher complication rate in our study is associated with implant removal failures. In our sample, the mean time to removal in cases of complications was 16 months, which is lower than the mean reported in the literature.

Among implants with incomplete removal, 2 flexible nails used to treat humeral fractures were not removed due to the corkscrew effect, 1 in the femur and 1 in the forearm, due to attempts to remove them being performed 16 months postoperatively. Among the cases of isolated screw removal, the screw broke in 2, allowing only partial implant removal. In the 4 cases of screws used in plate fixation, we were successful in removing the plate, but some screws broke, resulting in incomplete removal.

Implant insertion usually occurs with a minimally invasive approach. However, removal surgery can often be challenging, requiring a larger incision than the initial one, which can lead to a higher rate of complications, such as infections in postoperative wounds. 5

Postoperative infection was the second most frequent complication in our sample. The infections were superficial, clinically treated with antibiotics, without the need for additional surgical procedures.

In our study, we observed 2 cases of refractures that occurred after removal of the intramedullary Kirschner wire used for forearm fixation, and another after removal of a Limb Reconstruction System (LRS) external fixator to treat an infected femoral pseudarthrosis, with a permanence of 5 and 7 months, respectively. These cases may be related to insufficient bony callus formation, and patients underwent additional surgical procedures to treat this complication. Therefore, the indication for removal should occur after the bony callus is formed and the medullary canal is completely remodeled, to avoid the risk of refracture after removal. 4

Scheider et al. 10 evaluated the risk of complications associated with removal of upper limb implants in a hospital setting, there were 449 cases with an overall complication rate of 17.1%, and a mean time to implant removal of 23.7 months.

The risk of complications does not seem to be the same across all body sites, with cases in the upper limb being less common. 5 In our study, a similar pattern of upper and lower limb removals was observed, with no statistically significant differences in complications between limbs.

Lieber et al. 4 analyzed the removal of flexible intramedullary nails in 384 patients and found a lower complication rate of 3.1%. The technical rigor during the initial surgery directly affects the removal procedure, increasing the risk of complications with improperly placed implants. In our study, 4 nails could not be removed (2 humeral, 1 femoral, and 1 forearm fracture); 1 wound dehiscence; 1 superficial infection; and 1 patient was slow to regain knee mobility after removal.

Regarding the removal of isolated screws, the complication rate in the total sample was 14%. The study by Zimmerman et al. 9 evaluated the removal of screws used in the fixation of distal tibial fractures with deviation after 2 years of postoperative follow-up, compared with cases of implant maintenance and its long-term repercussions. Implant removal was performed in 17 patients and none had postoperative complications. Despite this finding, this study highlights that many procedures had difficulties and that they are not entirely benign, given the risks involved, requiring proper alignment with the child's family and/or caregivers. 11

Rehm et al. 12 evaluated implant removal in femoral diaphyseal fractures and observed a risk of refracture at a mean of 11 months after the procedure. In the removal of plates and screws, no refractures occurred. We had 7 complications among 41 removals, 3 screws were broken and not removed, 3 infections, and 1 patient reported discomfort after removal.

Elective implant removal should be considered, observing the risks and benefits involved with the procedure, due to its high cost potential neurovascular damage, implant breakage, infection, new or refractory complex regional pain syndrome, among other possible negative outcomes. 5 9 Therefore, there must be consensus among the surgeon, family members, and/or caregivers regarding the real need for the indication, as well as a warning about possible complications that may arise from it.

Our study has limitations, including its retrospective design that presents a high degree of heterogeneity in the sample, with different types of materials, surgical sites, and complications. The indication for implant removal did not follow a consistent pattern, and the preference of surgeons in charge was a factor to be considered. New multicenter studies to evaluate postimplant removal complications are essential to assist the orthopedic community in better decision-making.

## Conclusion

Implant removal in pediatric orthopedics is not without complications, with a 11% rate being found in this study. Complete removal failure, superficial infections, and refractures were the most common ones. The procedure should consider the risks and benefits involved and require consensus among family members and surgeons. Multicenter studies are suggested to expand knowledge on this topic.
